# DEMQOL and DEMQOL-Proxy: a Rasch analysis

**DOI:** 10.1186/s12955-017-0733-6

**Published:** 2017-08-22

**Authors:** A. A. Jolijn Hendriks, Sarah C. Smith, Theopisti Chrysanthaki, Stefan J. Cano, Nick Black

**Affiliations:** 1 Department of Health Services Research and Policy, London School of Hygiene & Tropical Medicine, 15-17 Tavistock Place, London, WC1H 9SH UK; 2 School of Health Sciences, Faculty of Health and Medical Sciences, University of Surrey, Guildford, Surrey, GU2 7XH UK; 3 Modus Outcomes, Spirella Building, Letchworth Garden City, SG6 4ET UK

**Keywords:** DEMQOL, DEMQOL-Proxy, Item analysis, Rasch measurement theory

## Abstract

**Background:**

DEMQOL and DEMQOL-Proxy are widely used patient reported outcome measures (PROMs) of health related quality of life in people with dementia (PWD). Growing interest in routine use of PROMs in health care calls for more robust instruments that are potentially fit for reliable and valid comparisons at the micro-level (patients) and meso-level (clinics, hospitals, care homes).

**Methods:**

We used modern psychometric methods (based on the Rasch model) to re-evaluate DEMQOL (1428 PWDs) and DEMQOL-Proxy (1022 carers) to ensure they are fit for purpose. We evaluated scale to sample targeting, ordering of item thresholds, item fit to the model, and differential item functioning (sex, age, relationship), local independence, unidimensionality and reliability on the full set of items and a smaller item set.

**Results:**

For both DEMQOL and DEMQOL-Proxy the smaller item set performed better than the original item set. We developed revised scores using the items from the smaller set.

**Conclusions:**

We have improved the scoring of DEMQOL and DEMQOL-Proxy using the Rasch measurement model. Future work should focus on the problems identified with content and response options.

## Background

DEMQOL and DEMQOL-Proxy [1–3] are well known and widely used patient reported outcome measures (PROMs) for measuring health related quality of life (HRQL) in people with dementia (PWDs). DEMQOL and DEMQOL-Proxy provide the means to assess HRQL at all stages of dementia severity. DEMQOL is self-reported by the PWD and is appropriate for use in mild/moderate dementia, DEMQOL-Proxy is proxy-reported by a family carer on behalf of the PWD and can be used at all stages of dementia. The two instruments are intended to be used together.

The original development of DEMQOL and DEMQOL-Proxy was grounded in strong methodology and robust psychometric principles [1, 3]. However, the use and application of PROMs is changing. In addition to their use in randomised controlled trials (RCTs) and other evaluative studies, there is a growing interest in the use of PROMs as part of routine monitoring of the quality of health and social care [4]. Routine use of PROMs provides an opportunity to help drive changes in how health and social care are organised and delivered [5] and to improve quality. Consequently, it is necessary to re-evaluate the measurement properties of PROMs to ensure that they are fit for these new purposes. To this end, in this paper we report the re-evaluation of the psychometric properties of DEMQOL and DEMQOL-Proxy.

Modern psychometric methods such as those based on Item Response Theory (IRT) [6, 7] and Rasch Measurement Theory (RMT) [8, 9] provide more stringent psychometric methods than traditional methods derived from Classical Test Theory (CTT). Since April 2009 PROMs data are routinely collected for some elective surgical operations in England [4, 10] and similar use is under consideration for other conditions, including dementia [11, 12]. Methodological work has been undertaken to apply IRT or RMT to the measures for routine use [13–18], but for measures of HRQL in dementia this has been limited. Rasch methods have been used with DEMQOL and DEMQOL-Proxy as part of the development of a health state classification system for DEMQOL-U and DEMQOL-Proxy-U [19]. No work has yet used Rasch methods to evaluate the whole set of DEMQOL/DEMQOL-Proxy items in terms of the overall score.

The measurement of outcomes in dementia is challenging. Cognitive impairment can make it difficult for a PWD to provide a reliable self-report on their HRQL and it may be necessary to rely on a proxy report from a family member. Yet, also proxy reports are methodologically challenging; proxies find it difficult to separate their own experience from that of the patient and for more subjective constructs, such as HRQL, PWD-proxy agreement is likely to be lower [20]. These challenges mean it is important that we apply the best available methodological techniques to ensure that the dementia specific outcome measures used in health services research, health care monitoring and individual clinical management are of the highest quality.

Modern psychometric approaches such as IRT and RMT have four advantages over CTT [21, 22]. The scores obtained are invariant, i.e. independent of the sampling distribution of the items used and locate items in a scale independent of the sampling distribution of the people in whom the scale is derived. They generate individual (rather than group) standard errors that clarify the degree of confidence in individual’s scores. Since scores are invariant there is greater potential to measure clinically meaningful differences. Finally, missing data can be dealt with more efficiently. Both IRT and RMT use mathematical (logit) models to improve the measurement properties of scores derived from questionnaires but they differ in the approach to data that do not fit the model: IRT tends to add parameters to the model whereas RMT investigates the data to identify why the misfit occurred. We used RMT to evaluate DEMQOL and DEMQOL-Proxy because the Rasch paradigm allows us to achieve interval scales, to identify potential anomalies with items and response scales, and at the same time, keep the conceptual framework on which the items are based central. This is important to ensure content validity and to produce scores that are clinically meaningful. Anomalies that are identified within the Rasch paradigm can help us to understand which particular items and response options are candidates for improvement. It also allows us to begin to build an evidence base about the extent to which instruments achieve invariant comparison. For example, differential item functioning (DIF) helps us to understand if any items are biased in favour of particular groups of the population. DEMQOL/DEMOQL-Proxy include a range of items about different aspects of daily life which arguably could also be affected by the aging process itself, gender roles and expectations and the deteriorating nature of dementia where eventually patients lose insight about their condition. Our analyses therefore enable us to understand which (if any) items are responded to differently by people of different ages, gender and severity.

## Methods

### Sample

The data were collected within a large study investigating the impact of Memory Assessment Services (MAS) on HRQL of PWDs [23]. Each of 78 MASs, geographically spread across all regions of the country and representative of all MASs in England, recruited up to 25 consecutive patients with suspected dementia who were attending for a first referral (either at the clinic or at a home visit) and their family carers (if present). Patients or carers with insufficient English to understand the consent procedure or study materials were not eligible for inclusion in the study.

### Instruments

DEMQOL consists of 28 questions and DEMQOL-Proxy consists of 31 questions, each assessed on a 4-point Likert-type response scale: *a lot*, *quite a bit*, *a little*, *not at all*. The questions were derived from five conceptual domains: health and well-being, cognitive functioning, daily activities, social relationships and self-concept [2] and with the exception of the emotion items all have the stem, “How worried have you been about…..”. There is also an additional overall quality of life question, answered on a 4-point scale: *very good*, *good*, *fair*, *poor*. The items are scored according to a standard scoring algorithm [24] to produce an overall score where higher scores represent better HRQL. See Smith et al. [1–3] for details on the development and CTT-based validation of DEMQOL and DEMQOL-Proxy.

### Data analysis

The use of modern psychometrics (IRT or Rasch methods) brings the opportunity to achieve more robust measurement by applying a mathematical approach to deriving scores based on a logit model. Modern psychometric methods are based on the relationship between a person’s location on the construct being measured (in this instance the level of their HRQL) and their probability of responding positively to each item. In contrast, traditional methods (such as Classical Test Theory) focus on the relationship between a person’s location on the construct and their observed total score on the scale. Thus, the analysis enables us to consider whether a measurement “ruler” has been successfully constructed. We evaluate this by considering whether i) response categories work as intended (threshold ordering); the items map out a continuum that is relevant to the people being measured (targeting); iii) the items work together (item fit); iv) responses to one item bias responses to another item (response dependency); v) performance is stable across relevant groups (differential item functioning (DIF); vi) items in the instrument represent a reliable unidimensional construct. The unique position of the Rasch paradigm is that when the data do not fit the model, the data (as opposed to the model) are scrutinised to determine the reasons why and to identify ways in which the items and/or response scales can be improved. Rasch based methods therefore provide a powerful set of diagnostic techniques which, although also generating more robust scores, can also highlight ways to improve the instruments in the future.

We conducted a Rasch analysis using RUMM2030 software to identify potential anomalies in the data indicating aspects of the instruments that were not working as intended [25]. Although all the items have the same 4-point Likert type scale, the unrestricted (partial credit) model was used as this was a diagnostic analysis and we wanted to evaluate whether each response scale was actually used similarly to each of the others.

All of the analyses were initially conducted for all items (28 for DEMQOL and 31 for DEMQOL-Proxy) and subsequently for a slightly smaller set of items that excluded the positive emotion items (23 items remaining for DEMQOL and 26 items remaining for DEMQOL-Proxy) as our early analyses [3] and preliminary work on this dataset (including parallel factor analysis – see Appendix) indicated that these items were conceptually different (trait items) and therefore represented a distinct dimension from the other items. We did not consider other reduced item sets because our aim was not to derive a shorter version of the scale. Rather we aimed to retain as many of the original scale items as possible and evaluate their performance. Because the sample was large, all estimates were based on the full sample, but to avoid type 1 error, the sample size was adjusted (*N* = 500), within the RUMM programme, before calculating significance tests (*p*-values).

#### Targeting

Scale-to-sample targeting concerns the match between the range of HRQL measured by the DEMQOL items (and DEMQOL-Proxy items) and the range of HRQL in the sample of PWDs. This was evaluated by comparing the spread of person and item (threshold) locations.

#### Ordering of item thresholds

We evaluated whether the response categories were working as intended by a visual inspection of the threshold map. As each item has four response categories, there are three thresholds per item, which should be ordered logically. Disordered thresholds can indicate where respondents have misunderstood or been unable to use response categories consistently. Collapsing (or re-scoring) the disordered thresholds can help to provide an indication of how response categories can be improved.

#### Item fit

The overall fit to the model was evaluated using chi-square. The fit of each item to the Rasch model was evaluated both statistically – fit residual within +/−2.5, chi-square statistic (Bonferroni corrected significance level) – and graphically (visual inspection of the item characteristic curve (ICC)). No single piece of information can confirm the fit of an item to the model and it is important therefore to consider all the evidence together.

#### Differential item functioning (DIF)

DIF is concerned with the extent to which different groups within the sample exhibit different scores for the same amount of the construct being measured. In this analysis for DEMQOL groups were defined as follows: PWD sex, PWD age group (quartiles), and disease severity (≥ 24 versus <24 MMSE or equivalent based on published cut offs indicating dementia). For DEMQOL-Proxy we additionally defined groups according to the sex and age group (quartiles) of the carer and relationship to the PWD (spouse, son/daughter, other). We used ANOVA to evaluate both main effects for these groups (uniform DIF) and interactions between these groups and the class intervals (non-uniform DIF). The presence of uniform DIF can be corrected by calibrating problem items separately for each level of the group (known as “splitting” items). Items showing non-uniform DIF may need to be investigated and/or removed from the item set.

#### Local independence

The extent to which each item was independent of the others was evaluated by examining the residual correlation matrix. Pairs of items where the residuals were correlated >0.3 were flagged. In the short term, the presence of response dependence can be corrected by considering each pair of dependent items to identify which is conceptually higher order. The lower order item is then calibrated (or “split”) by each level of the higher order item [26]. This avoids the need to remove items and further compromise content validity.

#### Unidimensionality

Item analysis by the Rasch model assumes unidimensional data. This was evaluated by prior factor analysis (Appendix) and principal components analysis (PCA) of the residuals to determine if there are any other identifiable dimensions in the data after the main “Rasch dimension” has been taken into account. If there is no interpretable pattern in the residuals then unidimensionality can be said to be supported [27]. Two subsets of four items were created from the highest and lowest loadings on the first principal component and a series of independent *t*-tests used to investigate whether the estimates for these two subsets differed significantly (percentage of individual *t*-tests outside the range ± 1.96). We computed Wilson 95% confidence intervals [28], as recommended by Brown, Cai, and DasGupta [29].

#### Reliability

Reliability was evaluated using the Person Separation Index (PSI), which is similar to Cronbach’s alpha. A value >0.7 is considered adequate.

### Rasch model based (logit) scores and their benefit

For both DEMQOL and DEMQOL-Proxy, we re-scored items with disordered thresholds (i.e. combining response categories as necessary). In addition, we resolved the items showing DIF (i.e. by splitting the relevant item and creating new items, one for each level of the person factor showing DIF) and/or local dependency (i.e. splitting the dependent item by the levels of the higher order item). We then generated Rasch model based scores (logits) for both resolved and unresolved versions. If the two versions were highly correlated, we retained the unresolved versions. The benefit of these scores over the raw scores was assessed by plotting them against the raw (original classically derived) scores. When the Rasch model based scores are different to the raw scores this will tend to give an ogive (“S”-shaped) curve.

## Results

### Descriptive characteristics of the sample

DEMQOL was completed by 1428 people with suspected dementia: 52% female, age range 42–98 years (mean age = 77.9, *SD* = 8.5) and 95% White or White British. DEMQOL-Proxy was completed by 1022 accompanying carers: 69% female, age range 16–94 years (mean age = 65.9, *SD* = 13.6), and 95% White or White British. Carers were predominantly the spouse (61%) or son/daughter (29%) of the PWD. Details of the sample are presented in Table 1

**Table 1:** Demographic characteristics of PWD and carer

Characteristics	*n* (%)
PWD:	
Sex	
Male	682 (47.8)
Female	746 (52.2)
Age	
< 73	352 (24.6)
73–78	334 (23.4)
79–83	352 (24.6)
> 83	390 (27.3)
Ethnicity	
White/White British	1343 (94.0)
Other ethnicity	78 (5.5)
Missing	7
Deprivation quintiles^a^	
1 – least deprived	349 (24.9)
2	299 (21.4)
3	280 (20.0)
4	253 (18.1)
5 – most deprived	219 (15.6)
Missing	28
Cognitive function^b^	
MMSE score < 24	701 (58.7)
MMSE score ≥ 24	494 (41.3)
Missing	233
Number of comorbidities^c^	
0	315 (22.1)
1	376 (26.3)
2	332 (23.2)
3	232 (16.2)
4 or more	173 (12.2)
Missing	6
Carer:	
Sex	
Male	312 (30.5)
Female	710 (69.5)
Age (y)	
< 57	245 (24.0)
57–67	254 (24.9)
68–76	272 (26.6)
> 76	251 (24.6)
Ethnicity	
White/White British	958 (95.2)
Other ethnicity	48 (4.8)
Missing	16
Relationship	
Husband/wife/partner	615 (61.0)
Son/daughter	295 (29.2)
Son/daughter-in-law	25 (2.5)
Sibling	14 (1.4)
Other relative	28 (2.8)
Friend	16 (1.6)
Neighbour	7 (0.7)
Other	9 (0.9)
Missing	13
Living with relative/friend	
Yes	683 (68.0)
No	321 (32.0)
Missing	18

^a^On the basis of the Index of Multiple Deprivation 2010 score

^b^Where MMSE score not available, ACE-III (≤ 82 vs. > 82), ACE-R (≤ 82 vs. > 82), MOCA (< 22 vs. ≥ 22), M-ACE (≤ 21 vs. > 21), KOLT (≤ 22 vs. > 22), or TYM (≤ 42 vs. > 42) score used based on established cut-offs for screening

^c^Selected from the following list of chronic conditions: heart disease (e.g. angina, heart attack or heart failure), high blood pressure, problems caused by stroke, leg pain when walking due to poor circulation, lung disease (e.g. asthma, chronic bronchitis or emphysema), diabetes, kidney disease, disease of the nervous system (e.g. Parkinson’s disease or multiple sclerosis), liver disease, cancer (within the last 5 years), depression or arthritis

*ACE-III* Addenbrooke Cognitive Examination-III, *ACE-R* Addenbrooke Cognitive Examination-Revised, *KOLT* Kendrick Object Learning Test, *M-ACE* Mini Addenbrooke Cognitive Examination, *MMSE* Mini-Mental State Examination, *MOCA* Montreal Cognitive Assessment, *PWD* person with dementia

### Overall fit to the model

For both DEMQOL and DEMQOL-Proxy the overall chi square statistic was non-significant (*p* = 0.99 and *p* = 0.11 respectively) suggesting that for both scales the data fit the model.

### Targeting

#### Original item sets (DEMQOL and DEMQOL-Proxy)

**Fig. 1:**
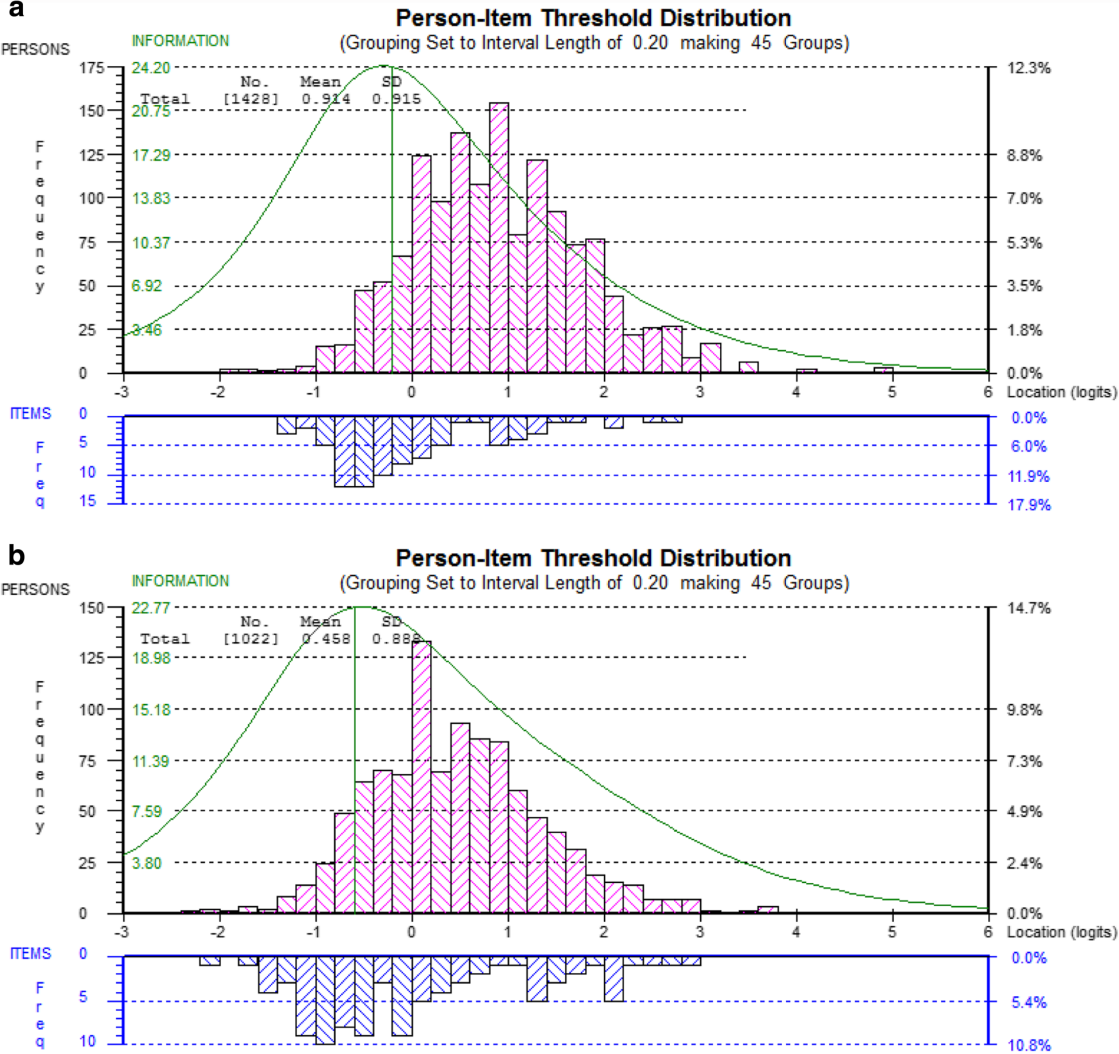
Person-item threshold location distribution for DEMQOL (**a**) and DEMQOL-Proxy (**b**)

For both DEMQOL and DEMQOL-Proxy, targeting of persons to item threshold locations could be improved (see Fig. 1a and b, respectively). In both cases, the spread of person locations (DEMQOL: *SD* = 0.915, DEMQOL-Proxy: *SD* = 0.888) covered the spread of item threshold locations well, though there was a lack of item thresholds at the high ends of the continuum.

#### Smaller item sets (DEMQOL and DEMQOL-Proxy)

**Fig. 2:**
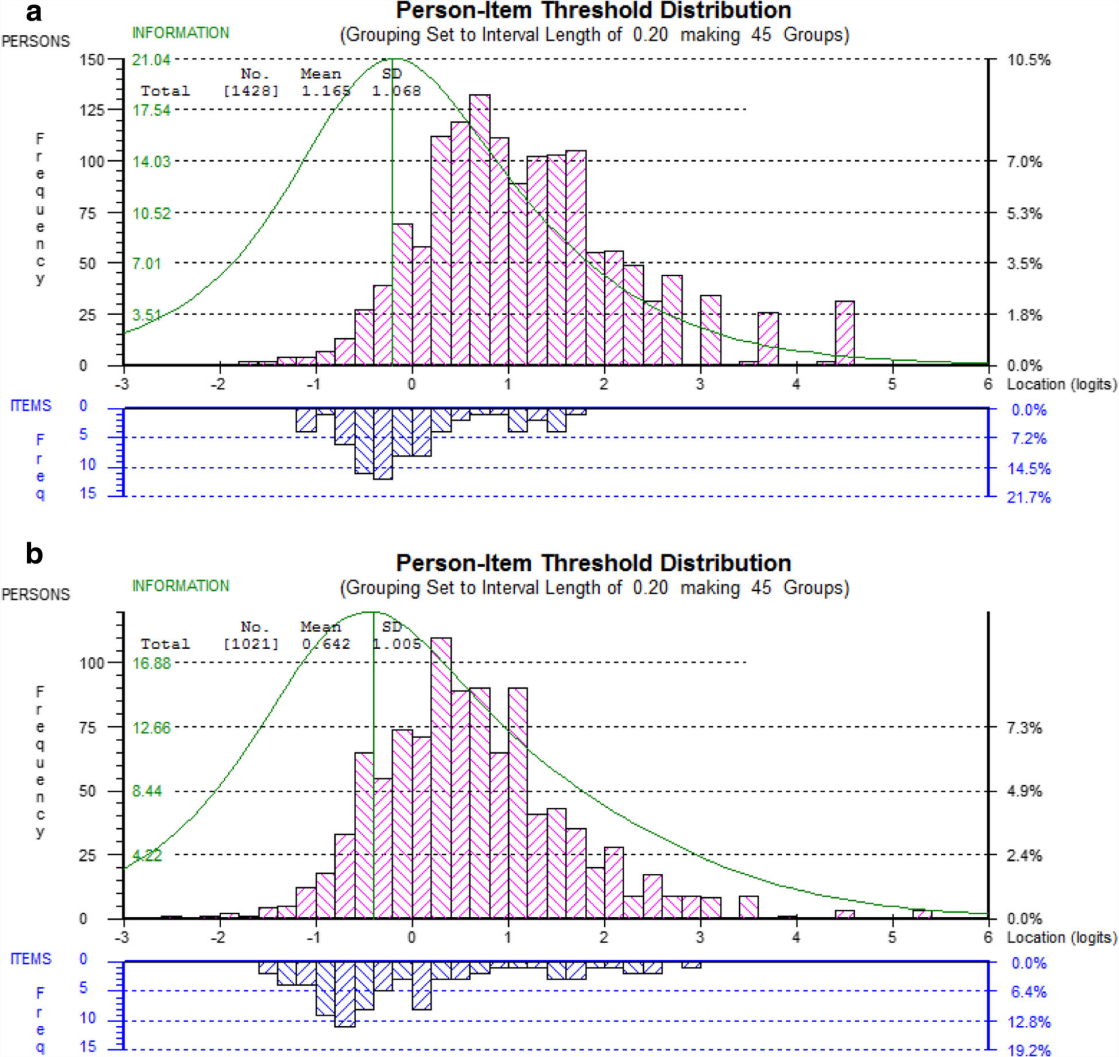
Person-item threshold location distribution for DEMQOL (23 items) (**a**) and DEMQOL-Proxy (26 items) (**b**)

For DEMQOL (23 items) (Fig. 2a) the range of item threshold locations is clearly smaller compared with the full set of items. For DEMQOL-Proxy (26 items) (Fig. 2b) the range of item threshold locations stayed almost the same because in contrast to DEMQOL, the highest located item thresholds included a wider range of items than just positive emotion items.

### Ordering of item thresholds

#### Original item sets (DEMQOL and DEMQOL-Proxy)

Five DEMQOL items and four DEMQOL-Proxy items showed response options not working properly (disordered thresholds). For DEMQOL these were having been worried about: a) not having enough company, b) how you get on with people close to you, c) getting the affection that you want, d) getting help when you need it, and e) getting to the toilet in time. For DEMQOL-Proxy these were having been worried about: a) keeping him/herself clean (e.g. washing and bathing), b) keeping him/herself looking nice, c) using money to pay for things, and d) looking after his/her finances. For all of these items we found that the middle two categories (“quite a bit” and “a little”) were not used as intended.

#### Smaller item sets (DEMQOL and DEMQOL-Proxy)

For DEMQOL (23 items), the same five items as in the original item set showed disordered thresholds. For DEMQOL-Proxy (26 items) we found one item less than in the original item set: having been worried about looking after his/her finances was no longer flagged. This may be due to the slightly smaller sample size (*N* = 1021) available for this analyses.

### Item fit

#### Original item sets (DEMQOL and DEMQOL-Proxy)

No DEMQOL or DEMQOL-Proxy items showed misfit to the model, considering the fit residuals, chi square values and the ICCs together (Table 2). However, four of the five DEMQOL positive emotion items (felt lively, full of energy, confident, cheerful, enjoying life) were among the items with the highest average threshold locations; the two highest (felt lively, full of energy) also showed large fit residuals (> +/− 2.5) and non-optimal fit to the ICC. We found this pattern largely replicated in DEMQOL-Proxy (Table 3), in particular for (felt) full of energy, lively and –to a lesser extent—cheerful.

**Table 2:** Diagnostic statistics for the original item set of DEMQOL (28 items)

Item	Location	Fit Residual	ChiSq	*p*	DIF	Item residual correlations	
						*r*	Correlation with item
1. Cheerful	0.480	0.452	2.08	0.99	*ns*	0.36, 0.27, 0.21, 0.20, 0.07, 0.04	3, 5, 10, 6, 12, 7
2. Worried or anxious	0.160	0.413	6.07	0.73	*ns*	0.22, 0.18, 0.10, 0.08, 0.01, 0.01	9, 4, 7, 14, 11, 12
3. Enjoying life	0.322	1.340	4.46	0.88	ns	0.36, 0.30, 0.25, 0.21, 0.06, 0.05, 0.04	1, 10, 6, 5, 12, 8, 7
4. Frustrated	0.352	−0.645	2.86	0.97	*ns*	0.18, 0.17, 0.15, 0.14, 0.07, 0.07	2, 11, 12, 9, 7, 13
5. Confident	0.578	1.129	5.46	0.79	*ns*	0.27, 0.21, 0.18, 0.18, 0.02	1, 3, 6, 10, 9
6. Full of energy	1.244	**4.014**	11.68	0.23	*ns*	0.53, 0.25, 0.20, 0.18, 0.07, 0.01	10, 3, 1, 5, 13, 12
7. Sad	−0.108	−1.561	6.79	0.66	*ns*	0.17, 0.15, 0.15, 0.10, 0.07, 0.04, 0.04, 0.01	12, 8, 9, 2, 4, 1, 3, 11
8. Lonely	−0.380	0.916	3.25	0.95	*ns*	0.39, 0.15, 0.14, 0.05, 0.04	20, 7, 12, 3, 9
9. Distressed	−0.563	**−3.047**	9.83	0.36	*ns*	0.22, 0.15, 0.14, 0.10, 0.07, 0.04, 0.02	2, 7, 4, 12, 11, 8, 5
10. Lively	1.340	**5.076**	17.35	0.04	*ns*	0.53, 0.30, 0.21, 0.18, 0.02, 0.01	6, 3, 1, 5, 12, 13
11. Irritable	−0.177	−0.343	2.89	0.97	*ns*	0.17, 0.15, 0.07, 0.04, 0.01, 0.01	4, 12, 9, 13, 2, 7
12. Fed-up	0.227	−2.171	6.69	0.67	*ns*	0.17, 0.15, 0.15, 0.14, 0.10, 0.07, 0.06, 0.04, 0.02, 0.01, 0.01	7, 4, 11, 8, 9, 1, 3, 13, 10, 2, 6
13. Things you wanted but couldn’t	0.633	1.526	7.15	0.62	*ns*	0.07, 0.07, 0.04, 0.04, 0.01, 0.01	4, 6, 11, 12, 10, 26
14. Forgetting happened recently	0.454	−0.907	3.93	0.92	*ns*	0.21, 0.18, 0.12, 0.12, 0.11, 0.08, 0.04, 0.04	17, 19, 15, 18, 2, 24, 27
15. Forgetting who people are	−0.197	**3.299**	8.56	0.48	*ns*	0.17, 0.15, 0.13, 0.13, 0.12, 0.08, 0.06	17, 16, 18, 19, 14, 24, 26
16. Forgetting what day it is	−0.034	**3.399**	7.23	0.61	*ns*	0.18, 0.15, 0.11, 0.07, 0.05, 0.03	17, 15, 14, 19, 18, 26
17. Thoughts being muddled	−0.046	−1.890	4.96	0.84	*ns*	0.28, 0.21, 0.18, 0.18, 0.17, 0.12, 0.07	19, 14, 16, 18, 15, 24, 27
18. Difficulty making decisions	−0.267	**−2.999**	7.47	0.59	*ns*	0.20, 0.18, 0.13, 0.12, 0.06, 0.05, 0.02, 0.01, 0.01	19, 17, 15, 14, 24, 16, 21, 23, 25
19. Poor concentration	0.117	−1.781	5.91	0.75	*ns*	0.28, 0.20, 0.18, 0.13, 0.08, 0.07, 0.05, 0.02, 0.02, 0.01	17, 18, 14, 15, 24, 16, 28, 25, 27, 21
20. Not having enough company	−0.572	−0.572	2.85	0.97	*ns*	0.39, 0.10, 0.09, 0.04, 0.03	8, 22, 25, 23, 21
21. Get on with people close to you	−0.567	−0.592	3.87	0.92	*ns*	0.46, 0.15. 0.10, 0.09, 0.03, 0.02, 0.02, 0.01, 0.01	22, 24, 23, 25, 20, 18, 27, 19, 28
22. Getting the affection you want	−0.663	0.959	5.59	0.78	*ns*	0.46, 0.11, 0.10, 0.10, 0.07, 0.02, 0.02, 0.01	21, 25, 20, 23, 24, 26, 28, 27
23. People not listening to you	−0.630	−1.712	4.10	0.90	*ns*	0.21, 0.10, 0.10, 0.09, 0.04, 0.02, 0.01	24, 21, 22, 25, 20, 28, 18
24. Making yourself understood	−0.490	−1.645	4.00	0.91	*ns*	0.21, 0.15, 0.12, 0.09, 0.08, 0.08, 0.07, 0.06, 0.04, 0.02, 0.01	23, 21, 17, 25, 15, 19, 22, 18, 14, 28, 26
25. Getting help when needed	−0.663	**−3.235**	6.27	0.71	*ns*	0.11, 0.09, 0.09, 0.09, 0.09, 0.06, 0.04, 0.02, 0.02, 0.01	22, 20, 21, 23, 24, 28, 26, 19, 27, 18
26. Getting to the toilet in time	−0.429	**2.661**	15.41	0.08	*ns*	0.06, 0.04, 0.03, 0.02, 0.02, 0.02, 0.01, 0.01	15, 25, 16, 6, 22, 28, 13, 24
27. How you feel in yourself	−0.170	**−5.376**	19.98	0.02	*ns*	0.22, 0.07, 0.04, 0.02, 0.02, 0.02, 0.01	28, 17, 14, 19, 21, 25, 22
28. Overall health	0.047	−2.102	7.97	0.54	*ns*	0.22, 0.06, 0.05, 0.02, 0.02, 0.02, 0.02, 0.01	27, 25, 19, 22, 23, 24, 26, 21

Fit residuals in bold are outside the acceptable range of +/− 2.5. Location = average item threshold location (logit). ChiSq = chi square value; *p* = chi square probability. DIF = differential item functioning; *ns* = non-significant. None of the chi square tests is statistically significant at *p* < 0.01 (Bonferroni corrected)

**Table 3:** Diagnostic statistics for the original item set of DEMQOL-Proxy (31 items)

Item	Location	Fit Residual	ChiSq	*p*	DIF	Item residual correlations	
						*r*	Correlation with item
1. Cheerful	0.154	−0.545	6.31	0.71	*ns*	0.42, 0.36, 0.28, 0.27, 0.22, 0.20, 0.11, 0.07	8, 6, 4, 11, 5, 10, 9, 7
2. Worried or anxious	0.469	−0.437	8.04	0.53	*ns*	0.34, 0.32, 0.28, 0.21, 0.17, 0.12, 0.04	3, 7, 5, 10, 9, 12, 6
3. Frustrated	0.410	−1.346	7.24	0.61	*ns*	0.34, 0.34, 0.31, 0.29, 0.25, 0.08, 0.05	2, 9, 10, 7, 5, 12, 6
4. Full of energy	1.439	**3.763**	19.31	0.02	*ns*	0.66, 0.28, 0.26, 0.19, 0.07, 0.06	8, 4, 11, 6, 31, 10
5. Sad	--0.485	**−2.889**	15.30	0.08	*ns*	0.39, 0.31, 0.28, 0.25, 0.22, 0.17, 0.15, 0.09, 0.06, 0.02	10, 7, 2, 3, 1, 9, 6, 11, 8, 28
6. Content	0.332	0.831	6.14	0.73	*ns*	0.36, 0.26, 0.23, 0.19, 0.19, 0.15, 0.07, 0.05, 0.04	1, 8, 11, 4, 10, 5, 9, 3, 2
7. Distressed	--0.564	**−3.147**	14.07	0.12	*ns*	0.32, 0.31, 0.29, 0.25, 0.23, 0.09, 0.07, 0.01	2, 5, 3, 10, 9, 6, 1, 16
8. Lively	1.299	**4.383**	23.41	0.01	*ns*	0.66, 0.42, 0.37, 0.26, 0.11, 0.06, 0.02	4, 1, 11, 6, 10, 5, 31
9. Irritable	--0.094	0.040	4.74	0.86	PWD sex PWD age Relation	0.34, 0.30, 0.23, 0.17, 0.17, 0.11, 0.07	3, 10, 7, 2, 5, 1, 6
10. Fed-up	0.126	**−3.633**	16.44	0.06	*ns*	0.39, 0.31, 0.30, 0.25, 0.21, 0.20, 0.19, 0.11, 0.10, 0.06, 0.03	5, 3, 9, 7, 2, 1, 6, 8, 11, 4, 28
11. Things to look forward to	0.470	**4.110**	26.13	0.00	*ns*	0.37, 0.27, 0.26, 0.23, 0.05, 0.01	8, 1, 4, 6, 28, 17
12. Memory in general	0.503	1.733	7.16	0.62	*ns*	0.18, 0.12, 0.09, 0.08, 0.04, 0.04, 0.02, 0.01	14, 2, 15, 3, 18, 31, 13, 29
13. Forgetting happened long ago	−0.587	**4.393**	19.07	0.02	*ns*	0.16, 0.06, 0.05, 0.04, 0.03, 0.02, 0.02, 0.02	15, 16, 27, 25, 14, 12, 20, 24
14. Forgetting happened recently	1.257	0.481	1.98	0.99	*ns*	0.30, 0.26, 0.26, 0.18, 0.18, 0.06, 0.03, 0.03, 0.01	15, 17, 18, 12, 19, 16, 13, 27, 26
15. Forgetting people’s names	0.523	**2.785**	10.10	0.34	*ns*	0.30, 0.17, 0.16, 0.12, 0.09, 0.09, 0.07, 0.07, 0.02, 0.01	14, 17, 13, 16, 12, 18, 19, 20, 27, 25
16. Forgetting where he/she is	−0.984	−1.341	5.45	0.79	*ns*	0.24, 0.18, 0.12, 0.09, 0.06, 0.06, 0.05, 0.03, 0.01	17, 18, 12, 19, 13, 14, 20, 23, 7
17. Forgetting what day it is	0.301	1.954	5.52	0.79	Severity	0.30, 0.26, 0.24, 0.17, 0.17, 0.04, 0.01	18, 14, 16, 15, 19, 23, 11
18. Thoughts being muddled	0.437	**−3.998**	23.64	0.00	*ns*	0.39, 0.30, 0.26, 0.18, 0.17, 0.09, 0.04, 0.03	19, 17, 14, 16, 20, 15, 12, 26
19. Difficulty making decisions	0.299	**−3.794**	16.87	0.05	*ns*	0.39, 0.19, 0.18, 0.17, 0.09, 0.09, 0.07, 0.03, 0.02	18, 20, 14, 17, 16, 26, 15, 27, 25
20. Making him/herself understood	−0.334	**3.519**	0.54	1.00	*ns*	0.19, 0.17, 0.07, 0.05, 0.05, 0.02, 0.02, 0.01, 0.01	19, 18, 15, 16, 27, 13, 25, 21, 24
21. Keeping him/herself clean	−0.860	1.746	7.57	0.58	*ns*	0.64, 0.13, 0.09, 0.05, 0.04, 0.03, 0.01, 0.01	22, 23, 24, 31, 29, 26, 20, 30
22. Keeping him/herself looking nice	−0.814	2.051	6.38	0.70	*ns*	0.64, 0.15, 0.08, 0.05, 0.05, 0.02, 0.01, 0.01	21, 23, 29, 24, 31, 30, 25, 27
23. Getting from the shops	−0.615	−1.029	2.99	0.96	*ns*	0.26, 0.15, 0.13, 0.11, 0.09, 0.06, 0.04, 0.03	24, 22, 21, 25, 27, 26, 17, 16
24. Using money to pay	−0.688	−0.991	5.64	0.78	*ns*	0.49, 0.26, 0.11, 0.09, 0.08, 0.05, 0.02, 0.01	25, 23, 27, 21, 26, 22, 13, 20
25. Looking after finances	−0.418	1.519	9.45	0.40	*ns*	0.49, 0.17, 0.11, 0.05, 0.04, 0.02, 0.02, 0.01, 0.01	24, 27, 23, 26, 13, 19, 20, 15, 22
26. Things taking longer	0.246	**−3.239**	12.97	0.16	*ns*	0.15, 0.15, 0.09, 0.09, 0.08, 0.08, 0.06, 0.05, 0.03, 0.03, 0.01	27, 30, 19, 29, 24, 31, 23, 25, 18, 21, 14
27. Getting in touch with people	−0.593	−1.469	5.18	0.82	*ns*	0.17, 0.15, 0.14, 0.11, 0.10, 0.09, 0.06, 0.05, 0.05, 0.03, 0.03, 0.02, 0.01	25, 26, 29, 24, 28, 23, 30, 13, 20, 14, 19, 15, 22
28. Not having enough company	−0.487	0.784	5.75	0.76	PWD sex Carer age Relation	0.10, 0.05, 0.03, 0.02	27, 11, 10, 5
29. Not able help other people	−0.593	0.668	3.07	0.96	*ns*	0.41, 0.14, 0.09, 0.09, 0.08, 0.04, 0.01	30, 27, 26, 28, 22, 21, 12
30. Not playing useful part	−0.368	**−3.119**	10.01	0.35	*ns*	0.41, 0.15, 0.11, 0.06, 0.02, 0.01	29, 26, 28, 27, 22, 21
31. His/her physical health	0.218	0.854	2.33	0.99	*ns*	0.18, 0.14, 0.08, 0.07, 0.05, 0.05, 0.04, 0.02	30, 29, 26, 4, 21, 22, 12, 8

Fit residuals in bold are outside the acceptable range of +/− 2.5. Location = average item threshold location (logit). ChiSq = chi square value; *p* = chi square probability. DIF = differential item functioning; *ns* = non-significant. None of the chi square tests is statistically significant at *p* < 0.01 (Bonferroni corrected)

#### Smaller item sets (DEMQOL and DEMQOL-Proxy)

None of the 23 DEMQOL items nor the 26 DEMQOL-Proxy items showed misfit to the model, considering the fit residuals, chi square values and the ICCs together (Tables 4 and 5). However, items that showed large fit residuals (> +/− 2.5) in the original item sets now tended to fit slightly better for both DEMQOL (23 items) and DEMQOL-Proxy (26 items).

**Table 4:** Diagnostic statistics for the smaller item set of DEMQOL (23 items)

Item	Location	Fit Residual	ChiSq	*p*	DIF	Item residual correlations	
						*r*	Correlation with item
2. Worried or anxious	0.349	0.886	3.54	0.94	*ns*	0.21, 0.15, 0.08, 0.02	9, 4, 7, 14
4. Frustrated	0.546	0.257	2.66	0.98	*ns*	0.15, 0.15, 0.14, 0.12, 0.06, 0.06	2, 11, 12, 7, 13
7. Sad	0.080	−0.255	6.58	0.68	*ns*	0.18, 0.15, 0.14, 0.08, 0.06, 0.01	12, 8, 9, 2, 4, 11
8. Lonely	−0.206	2.222	5.59	0.78	*ns*	0.40, 0.15, 0.15, 0.04	20, 7, 12, 9
9. Distressed	−0.391	−2.395	7.04	0.63	*ns*	0.21, 0.14, 0.12, 0.10, 0.05, 0.04	2, 7, 4, 12, 11, 8
11. Irritable	0.007	0.361	1.66	1.00	*ns*	0.15, 0.14, 0.05, 0.02, 0.01	4, 12, 9, 11, 7
12. Fed-up	0.425	0.361	2.20	0.99	*ns*	0.18, 0.15, 0.14, 0.14, 0.10, 0.04	7, 8, 4, 11, 9, 13
13. Things you wanted but couldn’t	0.829	**4.077**	12.51	0.19	*ns*	0.06, 0.04, 0.02, 0.01	4, 12, 11, 26
14. Forgetting happened recently	0.654	−1.100	4.43	0.88	*ns*	0.15, 0.12, 0.08, 0.06, 0.06, 0.02	17, 19, 18, 15, 16, 2
15. Forgetting who people are	−0.043	**2.511**	6.68	0.67	*ns*	0.11, 0.10, 0.08, 0.07, 0.06, 0.02, 0.02	17, 16, 18, 19, 15, 24, 26
16. Forgetting what day it is	0.135	**4.339**	11.45	0.25	*ns*	0.12, 0.10, 0.06, 0.03, 0.02, 0.02	17, 15, 14, 19, 18, 26
17. Thoughts being muddled	0.126	−2.376	8.27	0.51	*ns*	0.22, 0.15, 0.13, 0.12, 0.11, 0.06, 0.03	19, 14, 18, 16, 15, 24, 27
18. Difficulty making decisions	−0.103	**−2.777**	8.08	0.53	*ns*	0.16, 0.13, 0.08, 0.08, 0.02, 0.02	19, 17, 14, 15, 16, 24
19. Poor concentration	0.298	−2.338	6.86	0.65	*ns*	0.22, 0.16, 0.12, 0.07, 0.03, 0.02	17, 18, 14, 15, 16, 24
20. Not having enough company	−0.415	−0.981	5.01	0.83	*ns*	0.40, 0.07, 0.07, 0.02	8, 22, 25, 23
21. Get on with people close to you	−0.422	−1.291	5.21	0.82	*ns*	0.41, 0.11, 0.06, 0.06	22, 24, 23, 25
22. Getting the affection you want	−0.525	0.328	6.63	0.68	*ns*	0.41, 0.07, 0.07, 0.07, 0.03	21, 20, 23, 25, 24
23. People not listening to you	−0.479	−0.989	5.73	0.77	*ns*	0.17, 0.07, 0.06, 0.04, 0.02	24, 22, 21, 25, 20
24. Making yourself understood	−0.337	−1.792	4.27	0.89	*ns*	0.17, 0.11, 0.06, 0.06, 0.03, 0.02, 0.02, 0.02	23, 21, 17, 25, 22, 15, 18, 19
25. Getting help when needed	−0.511	**−2.833**	5.07	0.83	*ns*	0.07, 0.07, 0.06, 0.06, 0.04, 0.03, 0.02	20, 22, 21, 24, 23, 28, 26
26. Getting to the toilet in time	−0.267	**3.543**	17.96	0.04	*ns*	0.02, 0.02, 0.02, 0.01	15, 16, 25, 13
27. How you feel in yourself	0.016	**−4.440**	14.24	0.11	*ns*	0.21, 0.03	28, 17
28. Overall health	0.232	−1.848	6.27	0.71	*ns*	0.21, 0.03	27, 25

Fit residuals in bold are outside the acceptable range of +/− 2.5. Location = average item threshold location (logit). ChiSq = chi square value; *p* = chi square probability. DIF = differential item functioning; *ns* = non-significant. None of the chi square tests is statistically significant at *p* < 0.01 (Bonferroni corrected)

**Table 5:** Diagnostic statistics for the smaller item set of DEMQOL-Proxy (26 items)

Item	Location	Fit Residual	ChiSq	*p*	DIF	Item residual correlations	
						*r*	Correlation with item
2. Worried or anxious	0.637	0.278	3.76	0.93	*ns*	0.33, 0.32, 0.29, 0.23, 0.16, 0.08	3, 7, 5, 10, 9, 12
3. Frustrated	0.574	−0.677	4.27	0.89	*ns*	0.34, 0.33, 0.33, 0.29, 0.26, 0.04	9, 2, 10, 7, 5, 12
5. Sad	−0.330	−1.455	7.01	0.64	*ns*	0.42, 0.32, 0.29, 0.26, 0.20, 0.06, 0.01	10, 7, 2, 3, 9, 28, 31
7. Distressed	−0.425	−2.095	6.48	0.69	*ns*	0.32, 0.32, 0.29, 0.27, 0.24	2, 5, 3, 10, 9
9. Irritable	0.052	0.919	3.07	0.96	PWD sex PWD age Relation	0.34, 0.32, 0.24, 0.20	3, 10, 7, 5
10. Fed-up	0.287	−1.249	3.05	0.96	*ns*	0.42, 0.33, 0.32, 0.27, 0.23, 0.08, 0.02	5, 3, 9, 7, 2, 28, 31
12. Memory in general	0.664	1.529	6.49	0.69	*ns*	0.13, 0.08, 0.04, 0.04, 0.03	14, 2, 3, 15, 31
13. Forgetting happened long ago	−0.467	**4.704**	19.16	0.02	*ns*	0.13, 0.04, 0.01	15, 16, 27
14. Forgetting happened recently	1.462	0.373	4.66	0.86	*ns*	0.26, 0.22, 0.22, 0.14, 0.13, 0.05	15, 17, 18, 19, 12, 16
15. Forgetting people’s names	0.684	**2.627**	8.42	0.49	*ns*	0.26, 0.14, 0.13, 0.10, 0.04, 0.04, 0.03, 0.03	14, 17, 13, 16, 12, 18, 19, 20
16. Forgetting where he/she is	−0.866	−0.539	5.87	0.75	*ns*	0.23, 0.16, 0.10, 0.07, 0.05, 0.04, 0.03	17, 18, 15, 19, 14, 13, 20
17. Forgetting what day it is	0.451	2.045	4.86	0.85	Severity	0.27, 0.23, 0.22, 0.14, 0.14	18, 16, 14, 15, 19
18. Thoughts being muddled	0.604	**−3.813**	25.07	0.00	Age	0.36, 0.27, 0.22, 0.16, 0.14, 0.04	19, 17, 14, 16, 20, 15
19. Difficulty making decisions	0.458	**−3.475**	15.15	0.09	*ns*	0.36, 0.16, 0.14, 0.14, 0.07, 0.05, 0.03	18, 20, 14, 17, 16, 26, 15
20. Making him/herself understood	−0.201	2.456	3.60	0.94	*ns*	0.16, 0.14, 0.03, 0.03, 0.01	19, 18, 15, 16, 27
21. Keeping him/herself clean	−0.744	2.423	7.96	0.54	*ns*	0.64, 0.10, 0.06, 0.04, 0.02, 0.02	22, 23, 24, 31, 26, 29
22. Keeping him/herself looking nice	−0.702	2.008	9.74	0.37	*ns*	0.64, 0.11, 0.05, 0.05, 0.02	21, 23, 29, 31, 24
23. Getting from the shops	−0.501	−0.922	3.61	0.94	*ns*	0.23, 0.11, 0.10, 0.07, 0.06, 0.03	24, 22, 21, 25, 27, 26
24. Using money to pay	−0.578	−1.365	5.59	0.78	*ns*	0.47, 0.23, 0.08, 0.06, 0.04, 0.02	25, 23, 27, 21, 26, 22
25. Looking after finances	−0.304	0.915	9.25	0.41	*ns*	0.47, 0.13, 0.07	24, 27, 23
26. Things taking longer	0.402	**−2.987**	13.39	0.15	*ns*	0.13, 0.11, 0.07, 0.07, 0.05, 0.04, 0.03, 0.02	30, 27, 29, 31, 19, 24, 23, 21
27. Getting in touch with people	−0.472	−1.684	6.39	0.70	*ns*	0.13, 0.11, 0.11, 0.08, 0.08, 0.06, 0.03, 0.01, 0.01	25, 26, 29, 24, 27, 23, 30, 13, 20
28. Not having enough company	−0.352	2.197	7.79	0.56	PWD sex Carer age Relation	0.10, 0.08, 0.08, 0.08, 0.06, 0.01	30, 10, 27, 29, 5, 31
29. Not able help other people	−0.472	1.136	5.80	0.76	*ns*	0.39, 0.14, 0.11, 0.08, 0.07, 0.05, 0.02	30, 31, 27, 28, 26, 22, 21
30. Not playing useful part	−0.231	−2.067	8.27	0.51	*ns*	0.39, 0.18, 0.13, 0.10, 0.03	29, 31, 26, 28, 27
31. His/her physical health	0.369	**2.762**	9.22	0.42	*ns*	0.18, 0.14, 0.07, 0.05, 0.04, 0.03, 0.02, 0.01	30, 29, 26, 22, 21, 12, 10, 5

Fit residuals in bold are outside the acceptable range of +/− 2.5. Location = average item threshold location (logit). ChiSq = chi square value; *p* = chi square probability. DIF = differential item functioning; *ns* = non-significant. None of the chi square tests is statistically significant at *p* < 0.01 (Bonferroni corrected)

### Differential item functioning (DIF)

#### Original item sets (DEMQOL and DEMQOL-Proxy)

None of the DEMQOL items showed significant main effects (uniform DIF) for PWD sex, age group or severity. Three DEMQOL-Proxy items showed significant main effects. The item “feeling irritable” showed a significant main effect for patient age (carers of younger people report more irritability), patient sex (carers of men with dementia report more irritability) and relationship to the carer (spouse carers tending to report more irritability). The item “worried about forgetting what day it is” showed a significant main effect for severity (carers of people with MMSE scores <24 tending to report more worry about forgetting what day it is). The item “worried about not having enough company” showed a significant main effect for patient sex (carers of women with dementia reporting more worry about not having enough company), relationship to the carer (other carers tending to report more worry about not having enough company) and carer age (general trend for younger carers to report more worry about not having enough company). There were no significant interactions for any of the groups by class intervals.

#### Smaller item sets (DEMQOL and DEMQOL-Proxy)

None of the 23 DEMQOL items showed significant main effects for PWD sex, age group or severity. Three of the 26 DEMQOL-Proxy items showed significant main effects (uniform DIF). The item “feeling irritable” showed a significant main effect for patient sex (carers of men with dementia reporting more irritability) and patient age (carers of younger people reporting more irritability) and relationship to the carer (spouse carers tending to report more irritability). The item “worried about forgetting what day it is” showed significant main effects for severity (carers of people with MMSE scores <24 tending to report more worry about forgetting what day it is). The item “worried about not having enough company” showed significant main effects for patient sex (carers of women with dementia reporting more worry about not having enough company), carer age (younger carers tending to report more worry about not having enough company) and relationship to the carer (carers who are not a spouse reporting more worry about not having enough company). There were no significant interactions for any of the groups by class intervals.

### Local independence

#### Original item sets (DEMQOL and DEMQOL-Proxy)

Four pairs of DEMQOL items showed local dependency; the correlations were 0.36 (felt cheerful/that you are enjoying life), 0.39 (felt lonely/worried about not having enough company), 0.46 (worried about how you get on with people close to you/getting the affection that you want) and 0.53 (felt full of energy/lively), respectively, see Table 2. Fourteen DEMQOL-Proxy items showed local dependency, with correlations ranging from 0.31 (e.g. felt frustrated/fed-up) to 0.66 (felt full of energy/lively), see Table 3.

#### Smaller item sets (DEMQOL and DEMQOL-Proxy)

In the smaller item set for DEMQOL two residual correlations >0.3 remained (Table 4): felt lonely/worried about not having enough company (0.40) and worried about how you get on with people close to you/getting the affection that you want (0.41). For DEMQOL-Proxy in the smaller item set we found 11 residual correlations >0.3 (Table 5). The largest ones were between felt sad/fed-up (0.42), having been worried about using money to pay for things/looking after his/her finances (0.47) and keeping him/herself clean/ looking nice (0.64); the large residual correlation between felt sad/fed-up was new.

### Unidimensionality

#### Original item sets (DEMQOL and DEMQOL-Proxy)

Neither the 28 items in DEMQOL nor the 31 items in DEMQOL-Proxy formed a unidimensional scale. The PCA/*t*-test protocol showed that for DEMQOL the two subsets of measurements differed significantly for 12.3% [10.7; 14.1] of the cases at the 5% level and for 3.0% [2.0; 4.3] of the cases at the 1% level. For DEMQOL-Proxy they differed significantly for 12.0% [10.1; 14.1] at the 5% level and for 3.0% [1.9; 4.7] at the 1% level.

#### Smaller item sets (DEMQOL and DEMQOL-Proxy)

The smaller set of 23 items in DEMQOL formed an acceptably unidimensional scale though the smaller set of 26 items in DEMQOL-Proxy were still not unidimensional. For DEMQOL the two subsets of measurements differed significantly for 7.1% [5.9; 8.6] of the cases at the 5% level and for 1.1% [0.6; 2.1] of the cases at the 1% level. This is marginally more than can be expected by chance alone and is satisfactory, taking into account the large sample size [30]. For DEMQOL-Proxy the two subsets of measurements differed significantly for 11.9% [10.0; 14.0] of the cases at the 5% level and for 3.0% [1.9; 4.7] at the 1% level.

### Reliability

#### Original item sets (DEMQOL and DEMQOL-Proxy)

For DEMQOL PSI = 0.90, for DEMOL-Proxy PSI = 0.91, suggesting that both instruments discriminate well among people in terms of their HRQL (i.e. high reliability).

#### Smaller item sets (DEMQOL and DEMQOL-Proxy)

The smaller item sets showed similar PSI statistics. For the smaller set of 23 DEMQOL items PSI = 0.87, and for the smaller set of 26 DEMQOL-Proxy items PSI = 0.91.

### Rasch model based (logit) scores and their benefit

We derived Rasch model based scores for the smaller item sets (23 items for DEMQOL and 26 items for DEMQOL-Proxy) because of their generally better performance. For DEMQOL, we re-scored the five items with disordered thresholds. In addition, we resolved the two items that showed response dependency. Person location estimates with and without resolving for response dependency correlated *ICC* = 0.99, therefore, we kept the original estimates.

For DEMQOL-Proxy, we re-scored three items with disordered thresholds. In addition, we resolved the 11 items that showed response dependency and the three items that showed DIF were split. Person location estimates with and without resolving for these issues correlated *ICC* = 0.97, therefore, we kept the original estimates.

**Fig. 3:**
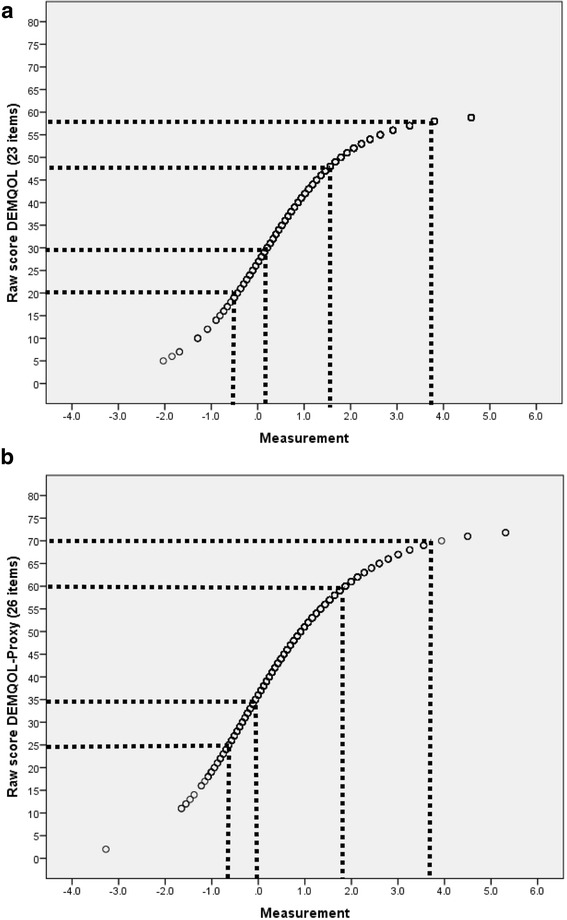
Relationship between raw scores and measurements (logits) for DEMQOL (23 items) (**a**) and DEMQOL-Proxy (26 items) (**b**)

The plots showing the benefit of the Rasch model based scores are shown in Fig. 3. The S-shaped curve clearly indicates that at the extremes of the distribution there is benefit from deriving the Rasch model based scores. For both DEMQOL (23 items) and DEMQOL-Proxy (26 items), a 10-point increase in terms of raw scores corresponds to a variable amount of increase in terms of logits, dependent on the person’s location on the raw score scale.

## Discussion

We have improved the scoring of DEMQOL and DEMQOL-Proxy using RMT and developed scores that can provide more robust and meaningful estimates of change and in addition are potentially appropriate for use with individual patients as part of the clinical decision making process. Neither of these were possible with the original CTT based scores. We have also identified a set of items about positive emotion included in the original questionnaires that do not have strong measurement properties. These items need further qualitative investigation to understand how they could be written more appropriately. In addition, we have identified that the response options may not be as easy for respondents to use as was originally reported. This also needs further qualitative investigation. Nonetheless using the new Rasch-based scores will potentially mean that at the group level evaluative studies will be able to report estimates of change that are more precise. Consequently, decisions based on these studies will be more robust and more easily justified. For example, while many researchers using CTT-based scores assume that points on the scale are equally distanced [31] (i.e. interval) in fact their level of measurement is merely ordinal. There is no information about the actual distances between points on the scale. Consequently change scores derived from ordinal scores (e.g. at baseline and follow up) can be difficult to interpret, as the distance between points on the scale may be different at baseline compared with follow up.

We are not advocating that a shorter version of DEMQOL/DEMQOL-Proxy should be administered. DEMQOL and DEMQOL-Proxy are already widely used and should continue to be administered in the standard form (28 items for DEMQOL and 31 items for DEMQOL-Proxy). The improved scores derived here can be calculated for existing datasets or for new data collected using the standard questionnaires. The three available scores for DEMQOL/DEMQOL-Proxy (original classically developed scores, DEMQOL-U /DEMQOL-Proxy-U and the new Rasch developed scores reported here) are based on the same conceptual framework [2]. Each score is a trade-off between measurement for a particular purpose and content validity. Future users should choose the measure appropriate for their purpose. The removal of the positive items in the Rasch scores does not mean that they are unimportant for HRQL in dementia, merely that in their current form and when combined with the other items in the scale, these items do not work as they were intended. Future qualitative work should investigate how these items could be improved to enable them to be retained in the scores.

Future work should also evaluate the effect of Rasch scoring (as described here) on the evaluation of change using DEMQOL and DEMQOL-Proxy. This could be retrospective using existing datasets or prospective. The s-shaped curve in Fig. 3 suggests that most difference between the original scores and the new Rasch scores will be seen at the extremes of the distribution. In a normal sample distribution, the effect of the Rasch scores at the group level may therefore be small. The Rasch scores however, provide added potential for use at individual level.

Our removal of the positive emotion items means there is item content that had been identified as important to PWD’s HRQL [2] that is not represented in the new Rasch scores for DEMQOL and DEMQOL-Proxy. A similar issue also occurred in the development of the original DEMQOL and DEMQOL-Proxy scales [1, 3] in that the items representing the domain of self concept were removed at the item reduction stage. Both of these are examples of the trade off that can sometimes occur between content validity and measurement properties. Our recommendation is that future work should prioritise investigation of the wording of both the “positive emotion” and “self concept” items to develop better ways of asking these questions within the questionnaire format. The targeting diagrams suggest that there are some parts of the continuum of HRQL not represented by items in the questionnaire, particularly at the “higher” end of the HRQL scale. Further qualitative work is needed to investigate these two issues. This further understanding of the construct of HRQL that underlies DEMQOL and DEMQOL-Proxy would also help to improve the apparent lack of unidimensionality of the items in the Rasch based DEMQOL-Proxy score.

Secondly, for some items, response options appear not to work as intended (i.e. disordered thresholds). The category probability curves and item threshold locations suggest that this may be because respondents do not distinguish between the two categories at the extremes of the response scale (i.e. between “a lot” and “quite a bit” and between “a little” and “not at all”). Alternatively, the labels of the two middle categories (“quite a bit” and “a little”) may not be meaningful. We have temporarily resolved this issue by re-scoring the items as dichotomous items by collapsing the two categories at either end of the response scale, but future work should investigate why for some items the response categories are not working.

Although this analysis improves the scores of DEMQOL and DEMQOL-Proxy, the progressive severity of dementia presents additional measurement challenges. In particular, with increasing severity there is likely to be a point where self-report of HRQL is no longer possible. Using DEMQOL-Proxy partially solves this problem, but it is well known that agreement of self and proxy reports is relatively low for subjective, non-observable constructs such as HRQL [20]. One of the possible reasons for lack of agreement between self- and proxy- reports is that the two different reporters use different constructs to define what we call HRQL. Further analysis using the Rasch model could build on these results to address this problem by equating the Rasch scores reported here for DEMQOL and DEMQOL-Proxy to determine if they can be placed on a single scale. Equating would evaluate whether DEMQOL and DEMQOL-Proxy can be placed on a common metric and therefore whether the two instruments actually measure the same construct. If this were the case, then DEMQOL-Proxy scores could be used with confidence even when self-report was no longer possible.

The current analyses were conducted on a large, representative sample of people attending a first appointment at MAS for suspected dementia [23]. The benefits of the Rasch analysis reported here are therefore based on data from people with relatively mild cognitive impairment and their carers. Future developments should investigate the effect on the model fit of including people with more severe cognitive impairment in the sample (particularly for DEMQOL-Proxy). Further, as the questionnaires are standardised instruments, developed in English, people without enough English language to understand and complete the questionnaire were excluded from the study. It was therefore not possible to investigate DIF by ethnic groups and we do not know whether and to what extent items within DEMQOL/DEMQOL-Proxy are affected by the ethnic status of the participants.

## Conclusion

We have established that DEMQOL and DEMQOL-Proxy can provide robust measurement of HRQL in dementia when scores are derived from analysis using the Rasch model. At the group level, estimates of change in evaluative studies will potentially be more precise than when using CCT-based scores and the Rasch based scores can also now be used at the individual level. This is an important improvement for making and justifying decisions. There still are a number of limitations. Further research into the anomalies that we have identified may further improve the two instruments in terms of breadth of content and optimizing answer categories. Furthermore, we need to investigate whether measurement properties are the same across ethnic groups and levels of dementia severity. In addition, in future work we will investigate whether DEMQOL and DEMQOL-Proxy can be placed on the same scale and if so a revised Rasch model based scoring algorithm can be produced. This would ensure that one could use DEMQOL-Proxy with confidence if a self-report on DEMQOL is no longer possible. Such an algorithm would be appropriate for use in both existing and new datasets.

## Figures and Tables

**Fig. 4:**
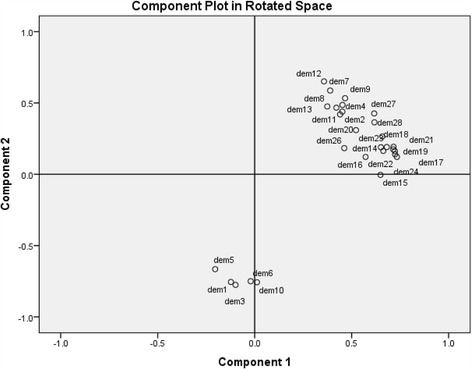
Plot of the factor loadings

## Appendix

### Results of the exploratory and parallel factor analysis

The factor analysis results for DEMQOL and DEMQOL-Proxy were highly comparable; therefore, we only present the results for DEMQOL. We performed an exploratory and parallel factor analysis based on principal components using the freely available software programme Factor 9.2 [32] to investigate the number of non-random components underlying the data. As recommended [32], the analysis was carried out on the polychoric correlations matrix because most DEMQOL items showed asymmetric univariate distributions with excess kurtosis. The results (Table 6) indicated at maximum [33] four content-related components (explaining more variance than parallel components extracted from 500 correlation matrices obtained from random permutations of the raw data). Only the first two components were sufficiently reliable (α ≥ 0.72, after rotation) to yield robust, replicable dimensions [34]. We computed α of the unrotated (α = 0.94 and α = 0.65) and varimax rotated (α = 0.83 and α = 0.75) components using the formulae published by Ten Berge and Hofstee [35].

**Table 6:** Exploratory and parallel factor analysis based on principal components

Item	Real-data eigenvalues	Mean of random eigenvalues	95 percentile of random eigenvalues
1	10.79144^a^	1.36547	1.41127
2	2.66292^a^	1.31325	1.35140
3	1.58357^a^	1.27487	1.30805
4	1.40969^a^	1.24164	1.26837
5	1.14704	1.21272	1.23873
6	0.93171	1.18559	1.20844
7	0.78525	1.15805	1.18129
8	0.72128	1.13369	1.15522
9	0.63767	1.11051	1.13066
10	0.59202	1.08769	1.10588
11	0.55460	1.06566	1.08420
12	0.53815	1.04397	1.06361
13	0.50040	1.02312	1.04151
14	0.48372	1.00303	1.02130
15	0.46846	0.98264	1.00091
16	0.45968	0.96292	0.98064
17	0.43176	0.94208	0.96037
18	0.39103	0.92086	0.93999
19	0.37195	0.90017	0.91964
20	0.35894	0.87983	0.89876
21	0.34018	0.85861	0.87875
22	0.32575	0.83670	0.85715
23	0.32086	0.81477	0.83436
24	0.28711	0.79187	0.81346
25	0.26326	0.76750	0.78862
26	0.25183	0.74040	0.76553
27	0.20604	0.71049	0.73984
28	0.18368	0.67193	0.70620

^a^Advised number of dimensions: 4

The first principal component was clearly dominant, explaining four times as much of the variance (10.79/28 = 38.5%) than the second component (9.5%). Table 7 shows the rotated component matrix. Four of the five positive emotion items loaded exclusively on a “Feelings” factor defined by positive and negative emotions; their secondary loadings on the first component defined by all other items (i.e. cognition, social relationships as well as negative emotions) were essentially zero. A plot of the factor loadings (Fig. 4) shows that the positive emotion items form a distinct, separate cluster.

**Table 7:** Rotated component matrix

Item	In the past week	Component	
		1	2
dem1	Felt cheerful	−.123	**−.755**
dem2	Felt worried or anxious	.452	.439
dem3	Felt enjoying life	−.099	**−.775**
dem4	Felt frustrated	.452	.486
dem5	Felt confident	−.204	−.665
dem6	Felt full of energy	−.021	**−.750**
dem7	Felt sad	.389	.587
dem8	Felt lonely	.374	.476
dem9	Felt distressed	.466	.533
dem10	Felt lively	.012	**−.757**
dem11	Felt irritable	.440	.420
dem12	Felt fed-up	.357	.651
dem13	Felt there were things you wanted to do but couldn’t	.421	.467
dem14	Worried about forgetting what happened recently	.651	.190
dem15	Worried about forgetting who people are	**.648**	−.004
dem16	Worried about forgetting what day it is	**.572**	.122
dem17	Worried about thoughts being muddled	**.724**	.140
dem18	Worried about difficulty making decisions	**.716**	.192
dem19	Worried about poor concentration	**.723**	.163
dem20	Worried about not enough company	.522	.310
dem21	Worried about how you get on with people close to you	**.714**	.175
dem22	Worried about getting the affection you want	**.663**	.164
dem23	Worried about people not listening to you	.681	.191
dem24	Worried about making yourself understood	**.733**	.122
dem25	Worried about getting help when needed	.658	.265
dem26	Worried about getting to the toilet in time	.462	.183
dem27	Worried about how you feel in yourself	.616	.427
dem28	Worried about your overall health	.617	.365

Primary loadings with a relatively negligible secondary loading are in bold; the primary loading is at least 3.73 (cotangent 15°) times as large as the secondary loading, therefore can be seen as factor-pure [36]

## Abbreviations

CTT: Classical Test Theory
DIF: Differential item functioning
HRQL: Health related quality of life
ICC: Item characteristic curve
IRT: Item Response Theory
MAS: Memory Assessment Services
PCA: Principal components analysis
PROMs: Patient reported outcome measures
PSI: Person Separation Index
PWD: Person with dementia
RCTs: Randomised controlled trials
RMT: Rasch Measurement Theory
SD: Standard deviation
